# TopicTracker – An advanced software pipeline for text mining on PubMed data: Bridging the gap between off-the-shelf tools and code based approaches

**DOI:** 10.1016/j.heliyon.2024.e36351

**Published:** 2024-08-15

**Authors:** Giovanni Spitale, Federico Germani, Nikola Biller-Andorno

**Affiliations:** a Institute of Biomedical Ethics and History of Medicine, University of Zurich, Zurich, Switzerland; b Institute for Biomedical Ethics and History of Medicine (IBME), Winterthurerstrasse 30, 8006, Zürich, CH, Switzerland

**Keywords:** PubMed data analysis, Text mining pipeline, Scientific literature processing, Customizable text mining, Automated literature review, Open-source text analysis, Reproducible workflow

## Abstract

**Background:**

The ever-increasing volume of academic literature necessitates efficient and sophisticated tools for researchers to analyze, interpret, and uncover trends. Traditional search methods, while valuable, often fail to capture the nuance and interconnectedness of vast research domains.

**Results:**

TopicTracker, a novel software tool, addresses this gap by providing a comprehensive solution from querying PubMed databases to creating intricate semantic network maps. Through its functionalities, users can systematically search for desired literature, analyze trends, and visually represent co-occurrences in a given field. Our case studies, including support for the WHO on ethical considerations in infodemic management and mapping the evolution of ethics pre- and post-pandemic, underscore the tool's applicability and precision.

**Conclusions:**

TopicTracker represents a significant advancement in academic research tools for text mining. While it has its limitations, primarily tied to its alignment with PubMed, its benefits far outweigh the constraints. As the landscape of research continues to expand, tools like TopicTracker may be instrumental in guiding scholars in their pursuit of knowledge, ensuring they navigate the large amount of literature with clarity and precision.

## 1 Background

The proliferation of scientific literature is a well-documented issue. To date (July 2024), PubMed indexes more than 37 million papers; and its growth rate was estimated to 4 % per year [1]. Even limiting the scope to very specific fields, the amount of indexed literature makes it impossible for a researcher to read that subset of publications in its entirety, or to keep up with the pace of new publications [2]: ‘nobody has a complete picture of the scientific knowledge in any given field at any time’ [1].

Text mining (TM) is an efficient way to extract organized and meaningful information from large amounts of structured or non-structured text – for instance PubMed records. In this sense, text mining can be used to analyze from a high-level perspective and map out much more information. TM will not replace human assessment and reading (for the time being), but it can complement and support it.

Multiple workflows and implementations of TM analysis on medical literature can be imagined: for example, one could extract keywords or MeSH terms used in papers resulting from a PubMed query, and use them to refine the search strategy, either including or excluding them from the query, in an iterative approach [2]. However, it can be that keywords do not entirely represent the content of a paper: in this sense, a viable workflow includes lemmatizing the content of the title and of the abstract (i.e.: reducing words to their non-flexed form using sets of rules that are language-specific) [3] and performing a frequency analysis of the lemmas, again, identifying strategies to expand or refine a query. Moreover, similar approaches could be implemented in the context of citation screening, ‘a phase of systematic review process that has attracted a growing interest on the use of TM methods to support it to reduce time and effort’ [4]. Queries capturing all the production of a research institution could be mined to identify the main topics on which said institution tends to publish; and the same process can be applied to journals, grants, or to individual authors. Vice versa, entries resulting from well-defined queries on specific topics could be mined to identify the institutions, researchers, or funders which are most relevant in that specific field, or to identify related and collateral areas of research.

Another TM approach to scientific literature is based on named entity recognition (NER). NER relies either on rule sets or on machine learning systems to identify and categorize entities such as people, numbers, dates, organizations. The use of NER techniques is rather common in TM bioinformatics, e.g. to identify, categorize, and eventually find co-occurrences of diseases, drugs, genes, or proteins [[5], [6], [7], [8]].

A multitude of tools exist to apply text mining techniques to scientific literature. Among the many, MaxQDA and NVivo, programs developed primarily for qualitative data analysis, include functions for dictionary-based entity recognition and for frequency analysis [9,10]; Leximancer, a software for quantitative content analysis, can perform co-occurrence analysis, frequency analysis, and produce semantic network maps [11,12]; WordStat can perform frequency analysis, thematic clustering, and proximity analysis [13]; Voyant Tools can perform frequency analysis, keyword-in-context, and thematic clustering [14]; bioNerDS can perform NER analysis [6]. An overview is provided in Table 1

**Table 1:** Summary of software commonly used for text mining.

Software	TM capabilities	License	Link
MaxQDA	frequency analysis, keyword-in-context, dictionary-based entity recognition	Proprietary	https://www.maxqda.com/
NVivo	frequency analysis, keyword-in-context, dictionary-based entity recognition	Proprietary	https://lumivero.com/products/nvivo/
Leximancer	co-occurrence analysis, frequency analysis, semantic network maps	Proprietary	https://www.leximancer.com/
WordStat	frequency analysis, thematic clustering, and proximity analysis	Proprietary	https://provalisresearch.com/products/content-analysis-software/
Voyant Tools	frequency analysis, keyword-in-context, thematic clustering	GPL3	https://voyant-tools.org/
bioNerDS	NER	Simplified 2-Clause BSD Licence	https://bionerds.sourceforge.net/

In addition to the software mentioned above, a plethora of packages dedicated to TM in R or in Python have emerged over the years.

However, all these tools have limitations with regard to their use for the TM analysis of PubMed data. None of them can be used to extract PubMed records in a clean, organized, and reproducible way; many require an expensive licence; many do not offer explicit documentation on their inner functioning (e.g.: lemmatizing strategies); none is specifically designed for the task mentioned above, so the implementation of TM workflows can result flimsy and difficult to automatize.

On the other hand, TM packages for R and Python offer a high degree of customizability, allowing the definition of clear, precise, transparent, and replicable workflows, but require a good command of the programming languages. This combination of factors impedes and slows down a broader use of TM techniques applied to scientific literature.

The pressing need to effectively harness the ever-growing repository of literature demands innovative solutions. Despite the wealth of tools currently available, they often lack in efficiency, transparency, or user-friendliness, leaving researchers torn between generic commercial tools and the steep learning curves of custom-coded solutions.

Based on these considerations, in the preliminary phase of designing our TM pipeline, we recognized a series of distinct needs and targets to be addressed. Foremost, there was an undeniable demand for a tool with a broad scope of application – within the PubMed data structure. Simultaneously, we identified a lack of standards in current TM tools, which often led to inconsistencies in output and replicability. Besides our views on open science and open source as a moral duty, it is also evident that there is a significant appetite in the community for an open-source tool, which would encourage collective development and transparency. Furthermore, the need for a context-specific tool that could be tailored to distinct research scenarios is clear. Yet this tool should also remain task-agnostic to cater to a broad variety of text mining objectives. A cornerstone of our design ethos was to make the pipeline easy to use, ensuring accessibility for both novice and expert users. Lastly, a feature we aimed to incorporate was the “code in view” concept. This meant users could peer into specific functionalities of the tool.

## 2 Implementation

In designing the architecture of our software, we rooted our decisions in the recognized needs of the text mining community. At the heart of our pipeline lies Python 3.8, an industry-standard programming language renowned for its versatility, readability, and vast ecosystem of libraries, making it a preferred choice over other languages. To ensure an easy to use yet transparent experience, we employed Jupyter as our primary interface. Jupyter offers the advantage of consolidating code and its output, fostering an environment where users can see, understand, and interact with every step of the analysis [15]. This “code in view” principle, embodied through the use of individual notebooks for specific tasks, encourages an inspectable and modular approach. Our pipeline leverages widely recognized and well-maintained libraries such as Pandas for data manipulation, Matplotlib [16] and Bokeh [17] for visualization, and SpaCy [18] for advanced natural language processing tasks. By building on these established foundations, we can ensure robustness while avoiding the pitfalls of “reinventing the wheel”.

## 3 Architecture

Given the foundational principles established, our software adopts a modular and task-specific architecture, compartmentalized across four Jupyter notebooks to facilitate ease of use and task-specific precision:

1. **Search and Download Notebook**: Serving as the entry point, this notebook aids users in constructing precise queries tailored to their research needs. Utilizing intuitive interfaces and prompts, users can specify search criteria and subsequently initiate the download of relevant PubMed entries. By decoupling this from other tasks, the notebook ensures that users can fine-tune their search without unnecessary complications.
2. **Content Analyser Notebook**: Transitioning from data retrieval to processing, this notebook is geared towards executing essential text mining operations. Here, users can perform various TM tasks such as tokenization, lemmatization, and frequency analysis. Leveraging the power of Python's robust libraries, this notebook provides a streamlined process to extract meaningful insights from the raw data acquired and do produce static visualization to start navigating the data.
3. **Interactive Data Exploration Notebook**: Building on the processed data, this segment of the software amplifies user understanding through interactive visualization. By employing the Bokeh library, it offers users the possibility to create their own visualizations and to delve deep into the analyzed content, facilitating a hands-on approach to data exploration and allowing users to glean patterns and insights more intuitively.
4. **Semantic Networks Notebook**: The final notebook shifts gears from textual analysis to network visualization. It further pre-processes the data generated by notebook 2, preparing structured table files suitable for import into Gephi [19], a renowned tool for network visualization. With this, users can construct and visualize semantic network maps, further extending the depth of analysis by discerning relationships, clusters, and patterns in the data.

**Fig. 1:**
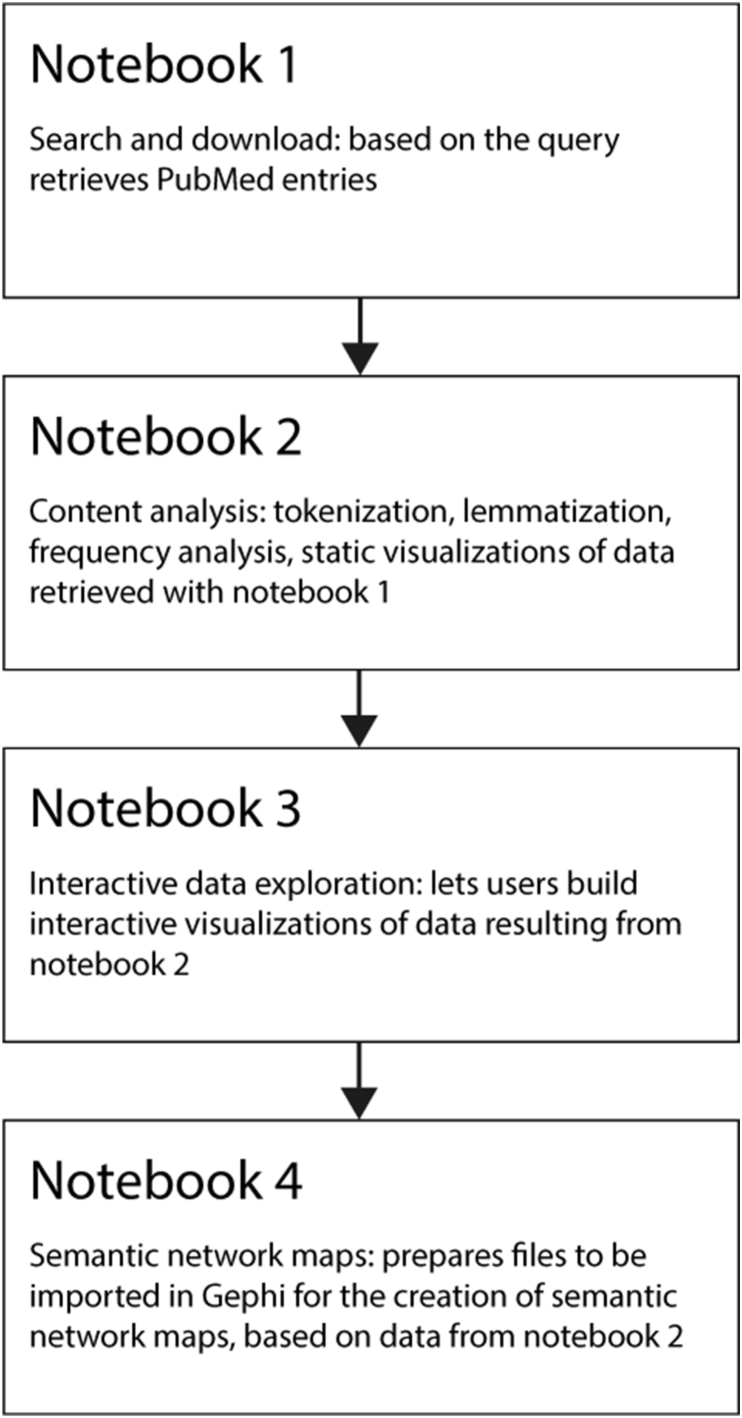
Flowchart of data across TopicTracker's notebooks.

This architecture, segmented yet interconnected, ensures that users can transition seamlessly across tasks, each notebook catering to a distinct phase in the text mining journey while maintaining cohesion and clarity throughout the process. The overall architecture and the data flow across notebooks is summarized in Fig. 1.

### 3.1 Data retrieval and extraction

The first notebook of our collection serves as a foundational tool for building precise PubMed queries, orchestrating the download of pertinent entries, parsing the acquired data, and saving the structured content into a.csv file. Upon initiation, the user provides a PubMed query. As a design consideration, PubMed limits the number of results to a maximum of 100,000 for each query. To address this, our software automatically segments the main query based on years. Individual queries are executed for each year, ensuring comprehensive coverage. Comprehensive logs are maintained for each segmented query, ensuring reproducibility.

**Fig. 2:**
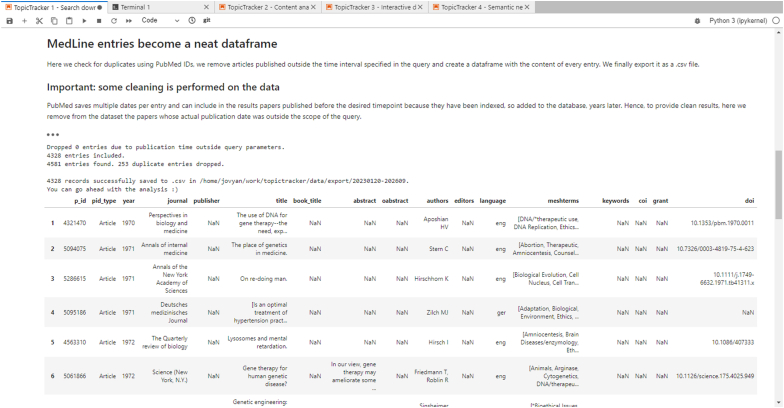
Interface of notebook 1 (data retrieval and extraction).

Based on the PubMed IDs retrieved from the query, this notebook queries the PubMed API to retrieve the MEDLINE record for each ID. The record is then parsed, and elements such as the ID itself, year of publication, journal, title, abstract, authors, MeSH terms, keywords, and conflict of interest statements, among others, are saved into a well-structured dataframe. The software then conducts a scan for duplicates using PubMed IDs. Notably, to maintain data integrity, our system addresses the intricacies of PubMed's multiple date-keeping by ensuring that entries added to the database years post their publication date and not in alignment with the query's temporal scope are pruned. The interface of notebook 1 is represented in Fig. 2.

Closing this notebook's functionality, a MedLine file is generated encompassing the retrieved entries. This resultant file stands ready for use, allowing researchers to seamlessly import references into reference management tools, such as Zotero, further facilitating the academic workflow.

Compared to direct PubMed downloads, TopicTracker offers significant advantages by integrating seamlessly into the TM process, organizing the data in tabular structures simple to access in the following steps, enhancing both velocity and efficiency. Its automated query segmentation and comprehensive data parsing ensure that researchers can swiftly obtain and organize large datasets.

### 3.2 Content analysis

The second notebook in this series delves deep into trend analysis over time. Leveraging the dataset produced using the first notebook, this notebook culminates in producing.csv files and.svg plots depicting trends across various entities.

**Fig. 3:**
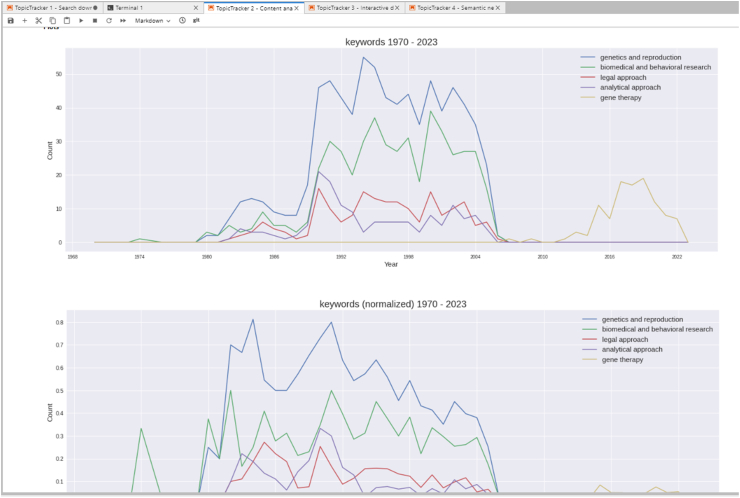
Automatic plotting of most frequent entities in the corpus (in this case, top 5 keywords in a corpus of records on 'ethics', 1970–2023).

Normalization emerges as a consistent methodology here, as it helps understanding the relative importance of a concept in the corpus. For most entities like keywords, MeSH terms, and authors, normalization is expressed as the count of a specific entity over the total number of papers. This would translate a normalized value of 0.1 for a keyword to indicate its presence in 10 % of the articles. However, this modus operandi differs for lemmas, given that a lemma might surface multiple times within a single paper. Hence, normalized values for lemmas should not be understood as percentages. The interface of notebook 2 is represented in Fig. 3.

The notebook delivers trends for keywords, MeSH terms, authors, and journals, outputting this data in.csv format and visualizing the top five entities in.svg plots. Delving deeper into titles and abstracts, lemmatization using SpaCy is employed to discern trends. Similarly, COI (Conflict of Interest) statements and their lemmatized trends are computed and represented. It's prudent to note that the COI section might benefit from a curated stoplist, filtering out common terms like “declare” or “interest” to ensure clarity in findings.

### 3.3 Interactive analysis

**Fig. 4:**
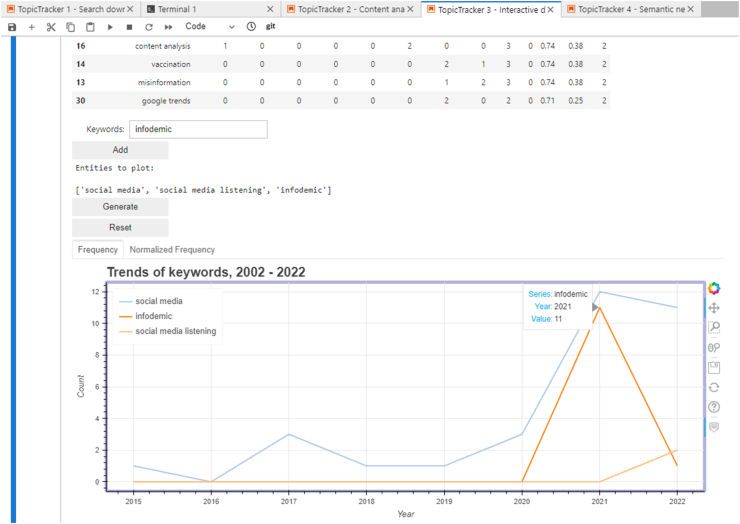
Interactive Bokeh interface for ordering and plotting the trends over time of selected keywords in a corpus (in this case, one of the preliminary queries later referred to in case study 1).

The third notebook stands as a powerful tool for immersive data exploration. Designed to work with the data processed by the second notebook, it offers a hands-on, customizable experience. One of the highlights of this notebook is the creation of an interactive visualization using the ‘bokeh' framework, which is renowned for its flexibility and user-friendly interfaces. The interface of notebook 3 is represented in Fig. 4.

Upon initializing this notebook, users are first prompted to choose the specific dataset they intend to delve into. Subsequently, preprocessed entities - keywords, MeSH terms, author, lemmas in title/abstract, lemmas in COI, journal - are available for selection. The data related to the chosen entity is presented in a table format, providing a comprehensive view of the entity details and sorting capabilities, allowing users to rearrange data based on different criteria.

Users can then handpick specific entities for visualization. Post selection, the system generates trendlines in ‘bokeh', delineating both the frequency and normalized frequency of these entities over time. These visual aids are not just static images; they are dynamic plots that users can interact with, delving deeper into specific data points. In essence, the third notebook not only empowers researchers with data but also grants them the autonomy to explore and visualize it as they deem fit.

### 3.4 Semantic network maps

The fourth notebook translates abstract data into detailed semantic network maps. Users can select a dataset processed with the second notebook and then compute a co-occurrence matrix. This matrix reveals the relationships between keywords in the corpus by assessing how frequently they appear together in the same paper. As a result, the software produces two distinct dataframes. The first, the 'nodes' dataframe, details the label and the weight of keywords. The second, the 'edges' dataframe, outlines the source, destination, and weight of connections between these labels based on their co-occurrence. This structured information then becomes the foundation for crafting a Gephi semantic network map.

**Fig. 5:**
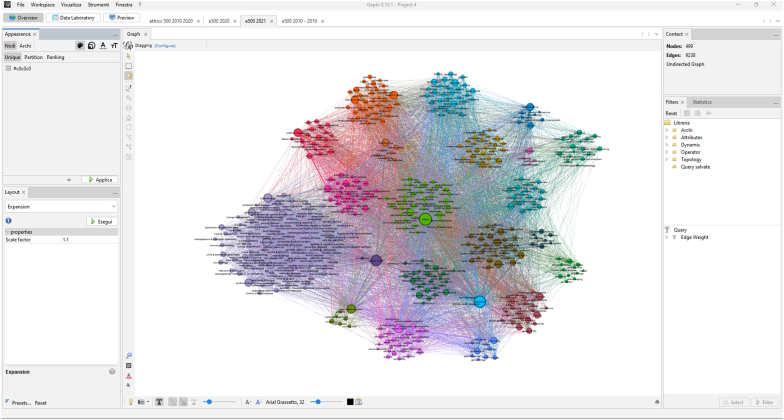
Importing nodes and edges tables generated with TopicTracker in Gephi, calculating modularity classes, and creating visual layouts (in this case, from the corpus later described in case study 2).

The process proceeds in a new Gephi workspace with the importation of nodes followed by the integration of edges, treated as non-directional connections. A pivotal analytical step then follows: the calculation of the modularity class, which allows the calculation of clusters of keywords based on their interconnectedness [20,21]. This modularity class plays a crucial role in determining the layout and the color-partitioning the nodes. Gephi's Circle Pack layout, using modularity class as the sole hierarchy, clusters similar nodes together, creating a visual hierarchy that's both intuitive and informative. The interface of a Gephi semantic network map built with data processed with notebook 4 is represented in Fig. 5.

In sum, in these semantic network maps, the prominence or relevance of a keyword within a corpus (so its normalized frequency) is visually indicated by the size of its node. The degree of interrelation with other individual keywords is depicted by the edges — the lines connecting one node to another. Moreover, distinct clusters or thematic areas (keywords’ modularity class) emerge through both the topological arrangement of these nodes and their color, providing a holistic view of the keyword landscape and its interconnections. The final map can be exported in multiple formats: as a static PDF for presentations and reports, or as an interactive Sigma.js [22] template for immersive online exploration.

The visual style and the methodology behind these visualizations are inspired by the work of Volodymyr Miz [23].

## 4 Discussion

Having explored the underlying architecture and capabilities of our software, we now transition into examining its real-world application. Two compelling case studies serve to underscore the software's efficacy and versatility. First, we delve into a literature review we are currently conducting for the World Health Organization (WHO) that focuses on the ethical dimensions of infodemic management — an exploration of how information, both accurate and misleading, spreads during health emergencies. Next, we present “Ethics 500,” a comprehensive semantic network map that showcases the 500 most influential keywords found in academic articles related to ethics, as indexed in PubMed.

### 4.1 Case study 1: query expansion (WHO infodemic)

As our institute (Institute of Biomedical Ethics and History of Medicine) at the University of Zurich is a World Health Organization (WHO) collaborating center, the authors of this paper were asked to support the work of an expert group (EG) focused on the ethical considerations surrounding social listening and infodemic management. The primary objective of the EG was to provide the WHO with informed counsel, enabling the creation of guidance and associated tools anchored in the ethical principles pertinent to social listening and infodemic management.

To fulfill this mandate, a thorough examination of existing literature was needed. In our pursuit, we employed TopicTracker to start our research with a basic query (v0):

"2002/01/01"[Date - Publication] : "2022/12/31"[Date - Publication] AND ("infodemic"[MeSH Terms] OR "infodemic"[All Fields] OR "infodemics"[All Fields] OR "social listening"[All Fields]) AND ("ethic s"[All Fields] OR "ethical"[All Fields] OR "ethicality"[All Fields] OR "ethically"[All Fields] OR "ethics"[MeSH Terms] OR "ethics"[All Fields] OR "ethic"[All Fields] OR "ethics"[MeSH Subheading])

Recognizing the richness and complexity of the field, we strategically mined the keywords from our initial search results, refining our approach iteratively [2]. This method allowed us to not only improve the scope and depth of our query but also to cast a wider net over the field of study. Remarkably, while query v0 surfaced only 34 papers—all of which published post the COVID-19 outbreak—our refined third iteration (v3) culminated in identifying 225 papers, ensuring we captured valuable insights from both the pre- and post-pandemic time, demonstrating the remarkable query-enhancing capabilities of TopicTracker. The query was later translated to the syntax of other databases, such as Scopus and Web of Science, in order to further broaden the coverage. All the queries, detailed methods, and the resultant datasets are accessible on this project's Open Science Framework (OSF) repository [24]. This case underscores the versatility and power of TopicTracker in guiding nuanced, expansive research endeavors.

### 4.2 Case study 2: Ethics500

The realm of ethics often finds itself adapting to the impact of contemporary global events. The COVID-19 pandemic stands as a testament to how real-world incidents can redirect and reshape the focus of ethical considerations. To visualize and understand these shifts, we leveraged semantic network maps to capture the landscape of keywords and their clusters within the field of ethics over different timeframes. The query to do so is simple:

"YYYY/MM/DD"[Date - Publication] : "YYYY/MM/DD"[Date - Publication] AND *ethic*[TiAb]

Our analysis focused on the most prominent 500 keywords in each corpus (hence ‘Ethics500’) and spanned three distinct periods: a pre-COVID baseline (2010–2019) (59961 papers), the year 2020 when the pandemic hit its stride (11091 papers), and the subsequent year, 2021, as the world grappled with the pandemic's ramifications (12075 papers). It is clear how approaching such a volume of literature with traditional reading approaches would be impractical. These maps vividly highlight the ebb and flow of themes in ethics. For instance, in the pre-pandemic phase, ‘public health'—with a weight of 0.01—was predominantly linked with areas like medical ethics, qualitative research, epidemiology, mental health, primary care, health policy, and preventive medicine. However, by 2021, the weight of ‘public health' surged to 0.05, reflecting its heightened importance. Moreover, its connections expanded and evolved to resonate with the pandemic-driven world, forming new links with thematic areas like protocols and guidelines, health economics, statistics & research methods, organization of health services, and infectious diseases, among others. Interactive Ethics500 semantic network maps are available online [25], and a static version is provided as supplementary material (Fig. S1). This case study underscores the power of semantic network maps in visually capturing the trends and trajectories of entire disciplines, providing high level but data-informed evidence on how tangible events can profoundly alter scholarly work.

### 4.3 Case study 3: addressing volatile ethical issues of Covid-19 with the Core Five Enduring Values list for health care professionals

In pursuit of constructing a dynamic list of ethical issues brought forth by the COVID-19 pandemic, we harnessed the versatility of TopicTracker to complement our theoretical framework (encompassing autonomy, privacy, equity, proportionality, and trust as core values) with a dynamic list of tangible issues where these values intersected with the Covid-19 pandemic [26]. This approach held particular significance as healthcare leaders grappled with multifaceted Covid-19 management challenges, necessitating a coherent overview that could steer appropriate adaptations. What set this analysis apart was its adaptability in fast-paced scenarios characterized by swift evolution. Our strategy involved running a set of five queries in TopicTracker, each focusing on the interplay between one of the Core Five Enduring Values and the COVID-19 pandemic.

Our efforts yielded results shedding light on the prevalence and content of discussions surrounding the Core Five Enduring Values in the context of the pandemic. While each of these values was deemed equally significant, our analysis revealed varying degrees of discussion, suggesting that certain areas warranted further exploration. Notably, topics related to autonomy, rights, and freedom, as well as trust and trustworthiness, emerged as widely discussed areas. Conversely, proportionality of measures received comparatively less attention, underscoring the need for more extensive research in specific domains [26]. As a content-wise example, our analysis highlighted that in the area of proportionality arguments tended to revolve around organizational ethics, public health restrictions, conflict of duties, border closures, and other related topics. The number of papers retrieved by each one of the queries at the time of the original study (May 2022) is reported in Table 2.

**Table 2:** Detailed results of the five ‘big five’ queries.

Query	Papers
The role of autonomy, rights and freedom in a pandemic	2215
Privacy vs. efficient and effective pandemic management	895
Equity, fairness and solidarity under conditions of resource scarcity	2659
Proportionality of measures: legitimation and procedures	55
Trust and trustworthiness	2311

As the content of these subcorpora was explored with TM, specifically with lemmatization and frequency analysis, this approach makes it possible to update the analysis swiftly and dynamically by re-running and re-analyzing the same queries.

Detailed outputs, as well as the original queries, are available for in-depth analysis [27].

### 4.4 Strengths

#### 4.4.1 Comprehensive workflow

Many software offerings provide piecemeal solutions, either focusing on data extraction, analysis, or visualization. TopicTracker, on the other hand, offers an end-to-end solution, integrating all stages from query construction to intricate visualization, ensuring users do not need to juggle between multiple platforms.

#### 4.4.2 Iterative query enhancement

TopicTracker allows for iterative query refinement. This feature ensures that the user can continually refine their search, obtaining an increasingly relevant dataset, as demonstrated in our WHO case study.

#### 4.4.3 Semantic network visualization

While some platforms provide rudimentary visualization tools, TopicTracker's integration with Gephi allows for the creation of advanced semantic network maps. This presents a bird's eye view of keyword relationships, offering insights that a simple frequency chart or word cloud cannot capture, as showcased in our Ethics500 case study.

#### 4.4.4 Time-bound analysis

TopicTracker's ability to segment data yearly is pivotal in tracking the evolution of topics over time.

#### 4.4.5 Integrated lemmatization

Utilizing advanced NLP tools like SpaCy, TopicTracker can delve deep into the linguistic structure of texts, providing lemma-based analysis. This is especially crucial when assessing trends based on the root meaning of words rather than just their surface forms, or just on keywords.

#### 4.4.6 Interactivity

TopicTracker's utilization of Bokeh for visualization ensures users aren't just passive viewers. They can actively engage, select, and drill down into the data, tailoring their exploration experience.

### 4.5 Limitations

#### 4.5.1 Database specificity

Currently, TopicTracker is primarily designed for PubMed. While this caters to a vast portion of biomedical literature, there are many other databases in diverse fields that remain untouched. However, it's worth noting that queries created for PubMed can be translated for other databases, making the software adaptable, albeit with some manual effort. Different databases often have distinct query syntaxes and metadata standards. This discrepancy can complicate the integration process and sometimes limit the set of variables common to all sources. For example, while fields like author, title, year, keywords, and abstract are generally consistent, database-specific fields such as MeSH terms are not.

Different databases use different APIs, necessitating custom implementations for each. For TopicTracker, we have developed a specialized Pythonic implementation of PubMed's API. With future funding, we aim to replicate this process for other databases, further enhancing TopicTracker's adaptability and utility across diverse research fields.

#### 4.5.2 URI length issue with PubMed's APIs

PubMed's APIs have a constraint wherein they accept queries via the Entrez system as strings appended directly to the URL. This design imposes a length restriction. As seen in our first case study, more comprehensive queries, like query v3, can lead to a 414 error indicating “URI too long.” This structural limitation, while not inherent to TopicTracker, affects its seamless functioning with PubMed. While the URI length issue poses a challenge, it's not insurmountable. We have devised a workaround to circumvent this obstacle. Researchers can directly search PubMed, retrieve the list of PubMed IDs representing the query results, and then feed these IDs into the TopicTracker (specifically, notebook 1). This method allows TopicTracker to download and process the corresponding entries, akin to how it manages shorter queries, leveraging notebooks 2 through 4.

#### 4.5.3 ‘As is’ software

It's worth noting that the software is provided ‘as is’ under a Creative Commons license. Due to the absence of dedicated funding, we are currently unable to offer continuous maintenance, support, or updates for TopicTracker. Interested third parties are free to rework the code according to their needs – or to fund us for doing so.

## 5 Conclusions

In the evolving landscape of academic research, efficient tools that allow for refined and informed exploration of literature are indispensable. TopicTracker, as delineated in this paper, embodies a comprehensive and nuanced approach to this need. Through its functionalities, from systematic querying of PubMed to intricate semantic network mapping, it provides researchers with profound insights into trends, clusters, and co-occurrences in their chosen fields. The case studies highlighted not only the applicability but also the real-world relevance of TopicTracker. Whether assisting global organizations like the WHO in navigating complex ethical terrains, or charting the dynamic shifts in the discourse of ethics pre- and post-pandemic, TopicTracker has proven a useful tool. In conclusion, TopicTracker provides a platform for deep-dived analysis, and encapsulates a step forward, we think, in how we understand, interpret, and leverage vast bodies of literature.

## Project name

TopicTracker.

## Project home page

https://doi.org/10.5281/zenodo.4520070.

## Operating system(s)

Platform independent.

## Programming language

Python 3.9.12.

## Other requirements

pandas = = 1.4.4; IPython = = 8.3.0; tqdm = = 4.64.0; matplotlib = = 3.5.3; bokeh = = 2.4.3; numpy = = 1.23.2; ipywidgets = = 8.0.1; spacy = = 3.4.1; wordcloud = = 1.8.2.2; shutils = = 0.1.0.

## License

CreativeCommons Attribution 4.0 International Public License.

## Any restrictions to use by non-academics

none.

## Ethics approval and consent to participate

Not applicable.

## Data availability statement

The code discussed in this article, along with a collection of toy datasets, can be accessed on the project's repository: https://doi.org/10.5281/zenodo.4520070.

For detailed documentation on the query expansion process outlined in case study 1, please visit this repository: https://osf.io/28d73/.

Data visualizations featured in case study 2 can be explored on the first author's personal website: https://www.giovannispitale.net/ethics500/.

To access the data utilized in developing the dynamic checklist described in case study 3, please visit this repository: https://zenodo.org/record/6726340.

## Funding

The authors did not receive specific funding for this work.

## CRediT authorship contribution statement

**Giovanni Spitale:** Writing – review & editing, Writing – original draft, Software, Data curation, Conceptualization. **Federico Germani:** Writing – review & editing, Writing – original draft, Conceptualization. **Nikola Biller-Andorno:** Writing – review & editing, Supervision, Conceptualization.

## Declaration of competing interest

The authors declare that they have no known competing financial interests or personal relationships that could have appeared to influence the work reported in this paper.

## List of abbreviations

TM: Text Mining
NER: Named Entity Recognition
API: Application Programming Interface
COI: Conflict of Interest
WHO: World Health Organization
EG: Expert Group
URI: Uniform Resource Identifier
URL: Uniform Resource Locator
