# Supplementary figures and images for: TopicTracker – An advanced software pipeline for text mining on PubMed data: Bridging the gap between off-the-shelf tools and code based approaches
Source: Heliyon. 2024 Aug 15;10(17):e36351. doi: 10.1016/j.heliyon.2024.e36351 (PMC11399583; doi:10.1016/j.heliyon.2024.e36351)

A semantic network map of the 500 most influential keywords in PubMed papers mentioning 'ethics'

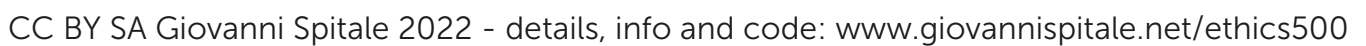

Supplement: Supplementary file 1 [file mmc1.pdf]
